# Macon Items

**Published:** 1898-10

**Authors:** 


### MACON ITEMS.

(FROM OUR SPECIAL CORRESPONDENT.)

Work on the annex of the Macon Hospital is going rapidly forward. Sandstone and pressed brick will be the materials used in its construction. The building will have two wards, having a capacity of twenty beds each, a modern operating-room, nurses' rooms, rooms for ward patients, kitchen, and a morgue.

The building is the gift of Mr. T. B. Gresham and his sister, Mrs. Mochin, of Baltimore, Md., and is a memorial to their father, who was once an honored citizen of Macon. It is to be known, therefore, as the "J. J. Gresham Memorial Building." The wards are intended for white patients only. The managers of the hospital are anxious to build at once a similar building for the colored patients.

Dr. H. McHatton, who has been for the past six weeks in the mountains of Carolina and North Georgia, has returned, and is full of fish stories.

At the meeting of the Macon Medical Society Tuesday evening, February 6, Dr. Olin J. Weaver read a most interesting article on "Drainage," which was discussed by Drs. Jackson, K. P. Moore, and Williams.

Dr. K. P. Moore reported a case of coccyalgia upon which he had operated. The most interesting feature of the case, and the one to which he called especial attention, was the presence of sugar in the urine, which was relieved by the operation. The doctor supposed the presence of sugar was a coincidence until he looked up the subject, and found that sugar in the urine was not an unusual complication of spinal bone lesions. The case was discussed by Drs. Jackson, Weaver, Shorter and Williams.

Dr. Moore also exhibited a very large ovarian cyst, the contents and sack weighing over forty pounds. The results in both the operations reported were perfectly satisfactory.

Dr. Williams exhibited a man who was stabbed July 30, in the left chest, just below the clavicle, the wound beginning half an inch from the sternum and extending four inches outwards and downwards. The second rib was cut in two just beyond the sternal attachment; the lung tissue was punctured, and there had been an extensive hematothorax and pneumatothorax. The bleeding was from the second intercostal artery, and was controlled by a plug of gauze thrust between the ribs in the shape of a pouch, then filled with absorbent cotton and pulled up tightly. It was impossible, owing to the exsanguinated condition of the patient, to hunt for and ligate the bleeding vessel. The patient made a slow recovery; still had a fistulous tract communicating with the pleural cavity. The drainage had been good, the collapsed lung had re-expanded, and the man was doing well. If in the course of a few months the fistula does not close, and there remains any pyopneumothorax, a resection of the ribs will have to be performed.

The case was discussed and examined by Drs. Shorter, Jackson, Moore, Weaver, Peete, and Gewinner.

